# The Strategy of Combining Antidepressants in the Treatment of Major Depression: Clinical Experience in Spanish Outpatients

**DOI:** 10.1155/2011/140194

**Published:** 2011-06-15

**Authors:** Luis M. Martín-López, Jose E. Rojo, Karina Gibert, Juan Carlos Martín, Lyli Sperry, Lurdes Duñó, Antonio Bulbena, Julio Vallejo

**Affiliations:** ^1^Institute of Neuropsychiatry and Addictions, Hospital del Mar, 08003 Barcelona, Spain; ^2^Department of Psychiatry, Hospital General Granollers Benito Menni CASM, 08400 Granollers, Barcelona, Spain; ^3^Statistics and Operation Research Department, Universitat Politècnica de Catalunya, 08034 Barcelona, Spain; ^4^Psychiatry Department, Hospital Universitari de Bellvitge, 08907 L'Hospitalet de Llobregat, Barcelona, Spain

## Abstract

*Introduction*. The combination of antidepressants is a useful tool in the treatment of major depression, especially in cases where there is a partial response to antidepressant monotherapy. However, the use of this strategy is a matter of controversy, and its frequency of use in clinical practice is not clear. The aim of our study is to assess the use of antidepressants combination in Spain by reviewing three databases used between 1997 and 2001. *Methods*. Databases pertain to patients who are study subjects of major depression treatment. These databases are a result of studies performed in Spain and in which 550 psychiatrists participated. The total studied sample was comprised of *N* = 2, 842 patients, aged over 18, fitting DSM-IV criteria for Major Depressive Episode. The percentage of patients who received more than one antidepressant and the types of combinations used was described. Subsequently, a comparative study between the group which received a combination of antidepressants (*N* = 64) and the group which received antidepressant monotherapy (*N* = 775) was performed. *Results*. 27.1% of patients were on antidepressive monotherapy treatment, and 2.2% were on combination therapy. In the comparison of patients on combination therapy and monotherapy, there were significant differences only in episode duration (*P* = 0.001). The most frequent combinations are SSRIs and tricyclic antidepressants. The active principle most widely combined is fluoxetine. *Conclusions*. The prevalence of use of antidepressant combination therapy is 2.2% of the global sample and 8.3% of treated patients. Other than duration of the depressive episode, no clinical characteristics exclusive to patients who received combination rather than monotherapy were found. Our study found that the most frequent combination is SSRIs + TCAs, also being the most studied.

## 1. Introduction

According to numerous authors [1–5], the combination of antidepressants is a useful tool in the treatment of major depression. It is a therapeutic strategy indicated especially in cases where response to antidepressant monotherapy is partial [3]. These situations arise regardless of the type of therapy used. In a review of 102 studies of patients treated with tricyclic antidepressants (TCAs), results indicated that on average, 51% of subjects responded favorably [6]. Similarly, in a review of 39 studies of major depression treated with SSRIs 47% of patients had a sufficient response to treatment. Given that 15% of the general population is prone to suffer a major depression episode at some point in their life [7], a substantial number of patients may require a combination of antidepressants to achieve a full response. However, indications, selection, and use of these combinations are a matter of debate. Algorithms such as the Texas Medication Algorithm project (TMAP) [8], Canadian clinical guidelines [9, 10], Hirschfeld sequencing [11], the “Sequenced Treatment Alternatives to Relieve Depression” (STAR*D) [12, 13], and McIntyre's algorithm [14] consider antidepressant combination therapy as second and third choice, to be applied only alternative monotherapies are proven ineffective. However, other groups, such as the Spanish Study Group for Combination of Antidepressants (SGCAD) [15], place it as first choice. Evidence-based medicine including case series [16, 17], open trials [18–20], and randomized double-blind controlled studies [21–23] suggest the efficacy of combination therapy for resistant depression or for rapid treatment. However, as relatively little remains known about use of this approach, in the last few years, several authors have called for studies describing the actual prevalence of combination therapy use through patient, general population, or physician surveys [24–26] as well as prescription studies [27–30].

The objective of our study is to analyze the frequency of use of combination antidepressants (ADs) versus monotherapy among patients with major depression in Spain, and to assess whether there are clinical or other predictors of the use of combination AD through the review of three databases corresponding to naturalistic studies performed in 1997, 2000, and 2001.

## 2. Material and Methods

### 2.1. Databases

Databases from three naturalistic studies performed by Organon S.A., in which psychiatrists from public mental health institutions and general hospitals at three sites across Spain participated between November 1997 and July 2001, were analyzed (see Table 1). The databases complied with a series of criteria.

- Participants met DSM IV criteria for major depression
- Information was available on any antidepressant drugs being taken by the patient prior to enrolment in the study.
- Objectives, design and methodology without bias effect, such as nonexclusion of patients who are under treatment.

Databases were compiled as baseline information in the context of studies assessing the efficacy of the antidepressant mirtazapine (see Table 1). At baseline, information was collected on sociodemographic characteristics, AD use, basic clinical characteristics, and psychiatric history of all eligible patients undergoing treatment for or diagnosed with major depression during the study timeframe, regardless of medication use. All patients provided informed consent, and ethical approval was obtained from each institution?

### 2.2. Study Subjects

The studied sample comprised **N** = 2,842  patients. All subjects included in this analysis were over the age of 18, fit DSM-IV criteria for major depression episode, and had a minimum score of 9 in the Hamilton Depression Rating Scale (HAMD17). Exclusion criteria included pregnant or breastfeeding women, concomitant presence of another psychiatric disorder such a bipolar disorder as the primary diagnosis, any clinically relevant and/or nonstabilized renal, hepatic, cardiovascular, respiratory, endocrine, or cerebrovascular pathology, as well as other serious, progressive, incapacitating or high-risk physical pathologies, and any other anomaly in the patient's health which could possibly interfere with the development of the study.

### 2.3. Study Variables

Sociodemographic and clinical variables were evaluated, including age, sex, marital status, previous history of depression, number of suicide attempts, characteristics of the current episode, duration of the episode, stressful life events, and the score on Hamilton's Depression Rating Scale (HAMD17). 

All drugs which were being taken by each patient at the start of the study were taken into account, including antidepressants, since the study did not exclude treated patients.

### 2.4. Data Analysis

A database was created by pooling databases from studies conducted in catchment areas in Spain, including 550 psychiatrists with a sample consisting of 2,842 patients (see Table 2). The percentage of patients who, at baseline, received more than one antidepressant and the types of combinations used was described. 

Subsequently, a comparative study between the group which received a combination of antidepressants (*N* = 64) and the group which received antidepressant monotherapy (*N* = 775) was performed. The Kruskall Wallis and Mann Whitney tests were applied to continuous quantitative variables (age, age of onset, Hamilton Scale score), and the Chi-square test was applied to categorical variables (e.g., sex, marital status, duration and level of depression, any previous suicide attempts, and previous depressive episodes). To assess the prevalence and patterns of use of combination AD therapy in patients with depression of shorter duration (e.g., perhaps more strongly related to severity, specific symptoms, or clinical history), we also conducted similar analyses among patients with episodes of of ≤ 6 months duration. 

For statistical analysis the SPSS 12.0 package was used. 

## 3. Results

The sample of 2,842 patients had mean ± SD age of 47.48 ± 14.50 years, 67.5% of them being women, and a predominant marital status of married (62.1%).

In Table 3 the clinical characteristics of each database and the global sample are described. The average age of onset was 37.34 (±14.53), 49.2% had a history of previous depressive episodes, 8.5% of patients had attempted suicide, and 50% presented with precipitating events. The most frequent duration was of 1 to 6 months, and 44.5% were characterized as being of moderate intensity.

Regarding the treatment being received when the interview took place, 27.1% were receiving antidepressant monotherapy, and 2.2% were receiving a combination, while 70.7% were not receiving antidepressant treatment.

In the comparison between patients receiving antidepressant combination therapy and monotherapy (see Tables 4 and 5), there were significant differences only in episode duration (*P* = .001) and not in age (*P* = .072), sex (*P* = .34), marital status (*P* = .058), age of onset (*P* = .86), suicide attempts (*P* = .21), mean Hamilton Scale score (*P* = .77), or level of depression (*P* = .16).

Though differences were not significant for the overall scale, the baseline Hamilton's Scale did show differences that the combination therapy group had higher scores for two items: item 13 (general somatic symptoms) and item 17 (weight loss) (*P* < .05 for both; see Table 4). 

Finally, the different types of combinations used according to drug family and active principle (see Table 6) were analyzed. The most frequent combinations were SSRIs and tricyclic antidepressants (*n* = 35 cases), followed by SSRIs and mianserine (4), TCAs with Dual antidepressants (4), SIRS and Dual antidepressants (4), and two SSRIs (3). 

The active principles most commonly combined were fluoxetine (*n* = 28 cases), amitryptilyne (22), paroxetine (17), and clomipramine (14). 

 Regarding the combination of SSRI + TCA, the most widely used combination was fluoxetine with amitryptiline, and the least used citalopram with clomipramine.

## 4. Discussion

Antidepressants combination treatments have become a popular method of treating refractory depression, enhancing therapeutic response in partial responders, and increasing the likelihood of more rapid response. Works from Nelson [6], Fava [31], Kelsey [32] and Shelton [33] support these strategies. Also, some researchers, Nelson [1], Besson [34], and Blier [23] have conducted studies using antidepressant combination from the beginning of the tretament in an attempt to obtain a more rapid onset of therapeutic action. The results from Besson [23] provide evidence that combination therapy from the beginning of treatment provides superior clinical effectiveness in the treatment of major depression.

The objectives of this study were to assess the real prevalence of the use of antidepressant combinations in the treatment of major depression in our country and analyze the clinical characteristics of these patients. 

Information on the use of combination antidepressant [35, 36] therapy is limited, and it remains uncertain whether the practice is widespread, and whether there are clinical indicators associated with its use. In this Spanish study, we found that among patients with major depression, the prevalence of combination therapy use was 8,3%%. The latter is comparable to rates varying from 1 to 5% reported in previous surveys conducted among subjects with major depression [25]. Rates of use were higher among patients with more persistent (>6 months) episodes of depression. Regardless of whether episodes were of longer or shorter (≤6 months) duration, use of combination therapy was not related to other clinical characteristics, including previous depressive episodes, patient sex, and level of depression. This suggests that inefficacy of monotherapy, rather than other *indicators* of clinical complexity, may be the primary determinant of selection of combination therapy. 

In the bibliographic revision, no studies of this kind are found. There are, however, other indirect methods to study the use of antidepressant combinations such as surveys and prescription studies, but the results obtained are all very different. 

In the surveys, the results obtained are varied, ranging from those that do not detect this tendency [35, 36] to those that do, with different results: 1% [24], 2% [37], 5% [25], and 15% [26].

The second method is based on studying prescriptions received by patients in medical consultations. Though it is closer to the primary care reality, sometimes the combination's indication is unknown (since the studies aim to detect polypharmacy), and results are also very varied: 2–5% [27, 28], 5% [38], 10% [39], and 24-25% [29, 30].

The second objective was to assess whether there are some clinical characteristics that predict patient use of combination AD treatment.

In the analysis of the clinical characteristics of patients receiving combination of antidepressants, the only difference found is that these patients present a disorder of longer duration. It is not possible to say they are more complex patients because, although their age is greater, with a greater number of previous affective episodes, more suicide attempts and chronicity of the disorder, results were not statistically significant. 

Patients receiving antidepressant combinations are not different from the point of view of intensity of depressive symptoms according to the analysis of global results from Hamilton's scale. Results also showed an unexpected consideration, which was obtaining a nonsignificant result in items 10 and 11, which refer to anxiety symptoms, since depression that progresses with anxiety requires higher dosage of antidepressants and is often more dependent on combination therapy [40].

The literature reviewed shows no studies that analyze the clinical and sociodemographic characteristics of the patient receiving combined treatment. The revised works tend to analyze prescription tendencies or perform open trials where the efficacy of the combination strategy with different types of molecules is demonstrated. 

However, some data obtained indirectly from the bibliographic revision can be contrasted with our study. 

As such, in clinical characteristics, patients who are combining antidepressants are defined as more complex due to their tendency towards chronicity [37] or to personality disorder comorbility [41]. These factors are indicators of combination [42].

Regarding the type of affective disorder, some studies aim to prove the efficacy of this strategy but highlight certain characteristics of these patients.

- Sethna [43], characterizes the patient who undergoes this strategy as a patient with a history of affective disorder of many years, with a tendency towards chronicity, dominant anxiety symptoms, without weight loss, early morning waking, or daily variation.
- Seth et al. [16] consider the factors of chronicity and resistance of the affective disorder as indicators for the use of this strategy.
- Mancini et al. [44] recommend the combination of SSRIs and noradrenergic antidepressants in depressed patients with obsessivoid symptoms.
- Bauer et al. [30] described that patients with more severe depression had a greater likelihood of receiving a combination of antidepressant.

The analysis of the antidepressants used shows that the combinations and drugs found in our study present similar behaviours except for slight differences, when compared with the revised literature. The high frequency of the combination of SSRIs and TCAs corresponds to a greater amount of scientific articles dealing with this subject. The reasons are the obvious greater use of these molecules and the prevalence of depression's monoaminergic theories in clinical practice, leading to the search for a full response when the use of SSRIs is accompanied by a partial response.

A more frequent use of fluoxetine is also detected, due to the fact that it is the first SSRI and most commonly used [45].

A difference is found in the type of TCA used. In our sample a greater use of amitryptiline is detected, while the literature shows that the most commonly used antidepressants are desipramine (not commercialized in our country), clomipramine, and nortriptyline. CAD is recommended to improve response and side effects (adjuntive therapy). Amitriptiline election would correspond better with the second indication.

Certain combinations are rarely advisable, such as the combination of two SSRIs, with little presence in the literature [46, 47] or totally inadvisable, such as the combinations of three and four antidepressants. In these cases, rather than speaking of combined therapies, the term to use would be polypharmacy. 

Finally, the study of older database might be interpreted as not representing the current use of antidepressants. It is true that there are new molecules such as new selective serotonin reuptake inhibitors (SSRIs), escitalopram, and dual antidepressants as duloxetine. However, the current prescription pattern is similar. In our study, we want to highlight the frequency and how to use combinations of antidepressants.

The arguments for this is as follows.

- The recent history of depression treatment is repetitive and highlights three periods. First, tricyclic and tetracyclic antidepressants (TCAs) were the first-line treatment choice treatment; in the second, selective serotonin reuptake inhibitors (SSRIs). Now, we also use “third generation” antidepressants (venlafaxine, duloxetine).
- In each of these periods (1965 and 1990), the combination of antidepressants becomes significant, especially in the decade of 90^ss,^ with the widespread use of SSRIs [2, 48] when the strategy of combining ADs became more widely known.

## 5. Conclusions

The use of the antidepressant combination strategy is an issue that has been treated in a fluctuating and controversial manner over the years. There are those who clearly support its use and those who question it [30].

The controversy heightens when the matter of this strategy's real use in clinical practice is posed. Further efforts to evaluate this strategy are called for [49].

In this study, a method different from those revised in the literature is applied, showing a prevalence of use of 2.2% in the global sample and 8.3% in treated patients.

In the analysis of the study's patients' characteristics, no traits are found to be exclusive of the patient who receives antidepressant combinations. Therefore, there are no criteria in the selection of this strategy. 

In regards to the type of combinations obtained in our study, SSRIs + antidepressants represent the most frequent option and the most studied and cited in the literature revised from the 90s. The years in which these studies were performed would also condition the choice of different molecules. This poses another question, since combinations acquire greater relevance with the appearance of SSRIs: which is better, to combine or use dual drugs? 

In our opinion, the strategy of combination of antidepressants is influenced by trends or tendencies in prescription patterns. Despite the controversies and limitations of different studies, the combination of antidepressants is justified as a second or third option, especially in cases where response is partial. 

The strengths of the study are that the data are representative of Spanish territory and offer results about the use antidepressant therapy in the practice.

Finally, we would like to comment on the limitations of our study, such as not learning about the psychiatrists who participated, which could elucidate the characteristics of the specialists who prescribe combinations, not knowing the dosage of the drugs and not being able to directly evaluate the efficacy of combination on a patient.

## Figures and Tables

**Table 1:** General characteristics of the three databases.

	Mar	Diana	Medas
Period of study	November 97	Septembert 98	February 01
	February 98	January 99	July 01

Objective of the study	Assess efficacy, onset of effect, and tolerance to mirtazapine in the treatment of major depression	Assess the efficacy and tolerance to mirtazapine in sleep and anxiety in major depression	Evaluate the clinical evolution of depression and somatic symptoms during the first three months of treatment with mirtazapine

Number of psychiatrists	300	150	100

Sample size	1261	877	704

**Table 2:** Catchement areas and psychiatrists.

Catchment areas	Mar	Diana	Medas
Castilla y Madrid	15.95%	18.46%	14.74%
Galicia y Asturias	13.62%	10.77%	11.58%
Euskadi	12.84%	12.82%	16.84%
Andalucía	20.24%	17.95	15.79%
Extremadura	—	—	3.16%
Canarias	0.78%	—	—
Levante	11.28%	12.31	10.53%
Catalunya y Baleares	25.29%	27.69%	27.37%

**Table 3:** Characteristics of each database and the global sample.

	MAR	DIANA	MEDAS	GLOBAL
Year of study	November 97	September 98	February 01	
	February 98	January 99	July 01	

Number of psychiatrists	300	150	100	550

Sample size	1261	877	704	2842

Mean (±) Age, years	47.1 ± 14.2	47.5 ± 14.4	48.0 ± 15.0	47.4 ± 14.5

Sex				
Women	68.2%	69.2%	66.0%	68.0%
Men	31.8%	30.8%	34.0%	32.0%

Marital status				
Unmarried	—	16.4%	19.2%	17.6%
Married	—	62.7%	61.2%	62.1%
Separated	—	11.2%	10.7%	11.0%
Widowed	—	9.0%	8.3%	8.8%
Unknown	—	0.6%	0.4%	0.5%

Age of onset	37.3 ± 14.5	36.4 ± 15	38.4 ± 13.8	37.3 ± 14.5

Previous suicide attempts (any)	7.8%	8.9%	8.9%	8.5%

Mean number of suicides/patient	0.14	0.15	0.14	0.14

Any previous depressive episode				
No	51.1	49.8	51.5	50.8
Yes	48.9	50.2	48.5	49.2

Other previous psychiatric pathologies	15%	27.9%	26.9%	21.7%

Unleashing factors	45.6%	50.4%	57.2%	50.0%

Episode duration (%)				
2 weeks	5.2	3.4	—	3.4
2 weeks-1 month	22.2	22.2	22.2	22.2
1–6 months	46.6	49.4	47.4	47.6
7–12 months	12.0	11.5	15.3	12.7
Over 1 year	14.0	13.6	15.2	14.2

Level of Depression				
Mild (7–17)	20.7	22.3	14.0	19.5
Moderate (18–24)	39.5	50.1	46.5	44.5
Severe (25–52)	39.7	27.6	39.6	36.0

Antidepressants taken				
No ADs	69.9	72.1	70.3	70.7
1 AD	27.4	25.8	28.2	27.1
ADs combination	2.7	2.1	1.5	2.2

**Table 4:** Comparative study of patients on one AD versus patients on AD combination.

	One AD	AD combination	Statistical Significance *P* ≤ .05	

Sample size	775	64		

Age	51.2 ± 14.35	53.1 ± 12.06	*Z* = −1.2	*P* = .072%
Sex			*χ* ^2^ = 0.91	*P* = .34
Women	72.7%	69.6%
Men	27.3%	30.4%

Marital status				
Unmarried	13.9%	8.6%	*χ* ^2^ = 3.58	*P* = .058
Married	62.8%	77.1%
Separated	11.5%	5.7%
Widowed	11.8%	8.6%
Unknown		0.6%

Age of onset	39.8 ± 15.1	40.3 ± 14.8	*Z* = −0.59	*P* = .86

Suicide attempts	10.7%	16.5%	*χ* ^2^ = 1.56	*P* = .21

Number of suicides/patient	0.19	0.36		

Previous depressive episodes			*χ* ^2^ = 0.85	*P* = .35
No	36.4	42.0
Yes	63.6	58.0

Other previous psychiatric disorders	25.0%	20.3%		

Unleashing factors	46.7%	56.5%		

Duration of episode (%)				
2 weeks	1.8	0.0	*χ* ^2^ = 16.0	**P** = .001
2 weeks-1 month	14.7	5.9
1–6 months	47.2	38.2
7–12 months	15.5	11.8
over 1 year	20.7	44.1

Hamilton Scale	21.69	21.69	*Z* = 0.287	*P* = .77

Level of Depression (%)				
Mild	18.5	18.7	*χ* ^2^ = 3.6	*P* = .165
Moderate	40.6	50.0
Severe	41.0	31.2

**Table 5:** Comparative study of Hamilton basal score one AD versus combination.

Hamilton D-17 items	One AD	Dt	Combination	dt	*P* ≤ .05
(1) Depressed mood	2.58	.77	2.76	.93	+
(2) Feelings of guilt	1.29	.88	1.24	.91	−
(3) Suicidal ideation	1.14	.96	1.11	1.03	−
(4) Early insomnia	1.49	.65	1.48	.72	−
(5) Middle insomnia	1.09	.63	1.05	.58	−
(6) Late insomnia	1.09	.75	1.08	.67	−
(7) Work and activities	2.37	.85	2.47	.94	−
(8) Retardation	1.06	.85	1.14	.85	−
(9) Agitation	1.14	.86	1.17	.77	−
(10) Psychic anxiety	2.08	.89	2.16	.82	−
(11) Somatic anxiety	1.86	.78	1.89	.76	−
(12) Somatic symptoms (gastrointestinal)	1.05	.64	.89	.65	−
(13) Somatic symptoms general	1.25	.55	1.17	.02	+
(14) Reduced libido	0.91	0.76	1.03	.78	−
(15) Hypochondriasis	1.30	.97	1.25	1.02	−
(16) Insight into illness	.31	.50	.19	.39	−
(17) Loss of weight	.70	.70	.41	.00	+
Total	22.67	5.91	22.34	5.52	−

**Table 6:** Combinations types.

Combinations types	Number
TCA + SSRI	35
TCA + DUAL ADs	4
SSRI + Mianserine	4
SSRI + SSRI	3
SSRI + DUAL ADs	3
TCA + TCA	3
SSRI + Trazodone	2
DUAL ADs + Mianserine	2
SSRI + Nefazodone	1
SSRI + TCAs + Nefazodone	1
Dual ADs + INA + Trazodone	1
SSRI + TCAs + INA	1
SSRI + TCAs(2)	1
SSRI(2) + Dual ADs	1
SSRI(3) + Dual ADs	1
RIMA + Trazodone	1
Total combinations	64

Dual AD:venlafaxine.

INA: Reboxetina.
